# Annotations

**Published:** 1888-03-10

**Authors:** 


### Annotations.

**Precept and Example at Paddington.**

In this country we like above all things to see a policy tried before we commit ourselves unreservedly to it. There have always been many, ourselves among the number, who have contended that the best way of dealing with the poor and the unemployed was to find them work, if possible, at living wages. When the distress was at its height in November last it was stated that Paddington was suffering acutely, and that something must be done promptly to avert worse consequences. Some of the principal inhabitants met under the presidency of Lord Randolph Churchill, Member for the Parliamentary Borough. The meeting resolved that as far as possible the Borough should deal with its own distress. This course was decided upon for the two-fold reason that it seemed to be best in itself, and that, if it should prove so in the result, the experience of Paddington might serve as a guide to other constituencies. The anticipations of the committee have been fully realised in each particular. A conference was held last Saturday at the residence of Mr. Aird, M.P., at which Lord Randolph Churchill again presided. A report of the Executive Committee was received, which dealt fully with the nature and extent of the distress, and the steps which had been taken to relieve it. The report showed that at the outset of their operations the committee had determined that the first step to be taken was to institute a thorough and exhaustive inquiry into the actual facts of the case, in order that they might know exactly what they had to deal with. The inquiry was made, and the number of unemployed proved to be practically what had been anticipated from previous knowledge. It was found that there were more than six hundred people who were willing to work, but unable find anything to do. A local fund was at once established, which before being closed amounted to £1,229. Mr. Beachcroft, a public-spirited inhabitant, offered to the borough as a free gift a large piece of ground extending to about eleven acres. To this the unemployed were sent, under efficient leadership, to convert it into a public recreation - ground. The experiment exceeded the most sanguine anticipations. The men set to work heartily; the amount collected proved quite sufficient to tide over the worst period of the distress; everybody is satisfied with the result; and Paddington has secured a large open space as a recreation-ground for its inhabitants for all time. The point to be emphasised is this, that one Parliamentary borough, dealing with its own distress in a practical way, has found the task not only well within its powers, but comparatively easy. The inference is that every other borough in London and elsewhere might probably, except in the case of the very poorest, find itself able to do the same thing. A large central fund, such as has heretofore been considered the best mode of dealing with distress, would seem to be unnecessary. If action be taken on the ines so successfully followed by Paddington a small fund to supplement the efforts of the poorer boroughs will probably in future be found amply sufficient; and each district, knowing its own wants, and getting such help as is indispensable, may be relied upon to do all that is necessary for itself.

**Cows for Cottagers.**

All plans, great and small, have been suggested for ameliorating the condition of the agricultural labourer; and it is quite possible that, from sheer bewilderment, he may take none of the counsels proffered him. "In the multitude of counsellors there is safety"—sometimes; difference of opinion, always. Perhaps in this matter the suggestions of the most unpretentious nature will prove the most valuable. The last of which we have heard is made by Mr. Evershed, that farmers should hire out cows to cottagers. It seems strange that in the country milk should not be one of the articles of most common consumption among all classes. In farmhouses it is so, but not in cottages. Labourers cannot afford to buy cows, and what milk they can get is bought from a farmer at a price not much less than what it would cost in town, and must, nevertheless, be accepted as a favour. More milk in the diet of our peasantry, to vary the monotony of bread and bacon, would undoubtedly improve their health; we cannot doubt that they would profit; the question is, would the farmers do so too? Mr. Evershed says they would, and brings figures to prove his statement. Farmers have tried the plan, and have prospered by it. It seems that a heifer will yield five times her weight in milk the first year, six times her weight the second, and seven times her weight the third, fourth, and fifth. The first year there is neither profit nor loss, the heifer just pays the cost of keep and hire; after that the increased quantity of milk brings a profit. There is certainly something very attractive, very pleasing to our English ideas of the fitness of things, in this plan of farmer and cottager mutually benefiting each other, instead of struggling more or less vainly to outdo a foreign competitor. Whatever be the necessities of our busy manufacturing towns, there is no reason why in our rural districts there should not survive that sense of mutual obligation and helpfulness which was the best part of feudalism. Everyone who has lived in the country knows how things not more important than this—the right to let a pig feed in a lane, or the possession of a plot of ground on which to rear a few fowls—may make the difference between prosperity and failure in a cottager's life; and if this plan of hiring a cow at a reasonable rate will be in any degree an economy to him, and at the same time profit the farmer to some extent, it is certainly worth trying.

**Still on Trial.**

The Provident Dispensary movement is still on its trial. In some towns it seems to work well, and to give satisfaction both to doctors and patients; in others its working appears to be unsatisfactory, and to please neither the one nor the other. The Manchester and Salford medical men, whilst fairly content with the system as a means of medical relief, are not satisfied with its rate of extension. The membership in that large centre of population ought, it is said, to be not less than 100,000; the actual figures are not higher than 20,000. This means either that a larger number of people go to the out-patient departments at the hospitals, or that private practitioners are more in request. On the other hand, a Birmingham doctor expresses decided dissatisfaction with the whole provident system as at present carried out. He thinks that many are drawn into its net whose little contributions would be better spent in buying food, whilst their medical relief should be the care of the parish surgeon. Many others who take advantage of it are well able to pay for the attendance of a private practitioner. These approvals and objections are, of course, mere repetitions of the *pros* and *cons* which have been heard from the beginning, and will continue to be heard until the end of time. But they are so numerous, and the whole subject is so vast and so important in its bearings, that it seems desirable for some general scheme to be formulated which will command the adhesion of the wiser and more statesmanlike of doctors on the one hand, and of the leaders of opinion among operatives on the other. If a complete scheme were formulated in the metropolis or other large centre of population which would satisfy the justice of the case all round, it could be modified to suit local circumstances, and yet give no occasion for such varieties of opinion and practice as now prevail. The Mutual Provident principle is so sound and so desirable in every way, that it is a pity it should fail in any locality for want of intelligence in the details of its working.

